# Profiling of chemical constituents of *Matricarla chamomilla* L. by UHPLC-Q-Orbitrap-HRMS and *in vivo* evaluation its anti-asthmatic activity

**DOI:** 10.1016/j.heliyon.2023.e15470

**Published:** 2023-04-24

**Authors:** Qian Li, Rahima Abdulla, Xuelei Xin, Guipeng Xue, Xiaolong Kang, Feicui Zhao, Haji Akber Asia

**Affiliations:** a Department of Medicine Research, Hospital of Chinese Medicine Affiliated to Xinjiang Medical University, Urumqi, 830000, People's Republic of China; b Xinjiang Key Laboratory of Processing and Research of Traditional Chinese Medicine, Urumqi, 830000, People's Republic of China; c CAS Key Laboratory of Chemistry of Plant Resources in Arid Regions, Key Laboratory of Xinjiang Indigenous Medicinal Plants Resource Utilization, Xinjiang Technical Institute of Physics and Chemistry, Chinese Academy of Sciences, 40-1, Beijing Road, Urumqi, 830011, Xinjiang, China; d Xinjiang Uygur Autonomous Region Evaluation and Inspection Center for Drug, Urumqi, 830000, People's Republic of China

**Keywords:** *Matricarla chamomilla* L., Anti-asthmatic activity, UHPLC, Mass

## Abstract

*Matricarla chamomilla* L. is native to European countries and widely cultivated in China, especially in Xinjiang. It has been used in Uygur medicine for the treatment of cough caused by asthma. In this study, UHPLC-Q-Orbitrap-MS was used to detect and identify the components from the active fraction of *M. Chamomile*, 64 compounds were identified by combining the standards, related literatures and mass spectrometry fragments, including 10 caffeoyl quinic acids, 38 flavonoids, 8 coumarins, 5 alkaloids and 3 other compounds. Furtherly, the anti-asthma activity of active fraction of *M. Chamomile* was investigated in OVA-induced allergic asthma rat model. The results showed that the number of EOS in Penh and bronchoalveolar lavage fluid (BALF) in the group of the active fraction of *M. Chamomile* was significantly lower than that in the model group. Besides, the active fraction of *M. Chamomile* can significantly reduce the IgE level and increased glutathione peroxidase (GSH-Px) in the serum of OVA-induced rats, and ameliorated OVA-induced lung injury. Hence, *M. Chamomile* could be used to treat asthma through their *in vivo* antioxidant and anti-inflammatory effects. This study explored the potential material basis of *M. Chamomile* for the treatment of asthma.

## 1 Introduction

Asthma is a heterogeneous disease characterized by chronic airway inflammation and is currently among the most common noninfectious diseases in children and adults, affecting approximately 334 million people worldwide [1]. The pathogenesis of asthma is complex, and well-recognized clinical mechanisms are the interaction of chronic airway inflammation and airway hyperreactivity [2]. These lead to variable respiratory symptoms such as wheezing, dyspnea, chest tightness and cough, which can reduced quality of life and even death prematurely [3]. Inflammation plays an important role in the pathophysiology of asthma. Currently, steroidal anti-inflammatory agents combined to bronchodilators are considered as common treatment for controlling asthma [4]. However, its efficacy is limited due to adverse effects and drug dependence or drug resistance. Recently, natural herbal plants and their active ingredients is gaining a lot of attention in the treatment of asthma due to their advantages of high efficiency, few side effects, and low cost. For instance, the extract of *Mandevilla longiflora* improves airway inflammation in amurine model of allergic asthma, which is rich in phenolic compounds and flavonoids [5]. Besides, plant polyphenols are active ingredients from Chinese herbal medicine or natural food, which have antioxidant, anti-allergic, anti-inflammatory; and previous experiments proved that resveratrol, genistin, luteolin and quercetin have anti-inflammatory effect with the characteristics of multiple targets, multiple links and comprehensive coordination [6]. Hence, many researchers have focused on polyphenol components derived from natural plants as complementary and alternative medicine to against asthma [7].

*Matricarla chamomilla* L., belong to Asteraceae family, is a native to European countries and and widely cultivated in China, especially in Xinjiang [8]. It has a long history as a spice and as a medical plant to be extensively served in food, cosmetics, and pharmaceutical industries. As a traditional Chinese medicine, *M. chamomilla* has been reported to have anti-inflammatory, warming the stomach and appetite, promoting digestion, reducing swelling, dispersing knots, antioxidant and anticancer activities, these effects may be associated with natural ingredients, such as flavonoids and phenolic acids [[9], [10], [11], [12]]. Besides, *M. chamomilla* is also one of the important components of Zukamu Granule, a prescription recordeded in the Pharmaceutical Quality Standard of the Ministry of Health of the People's Republic of China (Uyghur Medicine Sub-volume), which has been proved to be used for the treatment of common cold or upper respiratory infection in clinical practice [13]. The anti-inflammatory effects of *M. chamomilla* are well established, and they are broadly used in the management of several respiratory diseases [10,11,14,15]. Thus, *M. chamomilla*, as a natural resource, are considered to have the pharmaceutical benefit of anti-asthma.

Based on the above, this study aimed to identify the potential active ingredients of *M. chamomilla* using liquid chromatography-mass spectrometry (UHPLC-Q-Orbitrap-HRMS) technology and evaluated for anti-asthmatic activity by using *in vivo* rat models.

## 2 Materials and methods

### 2.1 Materials and reagents

Sulfoxide (DMSO) were purchased from Sigma-Aldrich (USA). Formic acid, methanol and acetonitrile for HPLC were obtained from Merck (Darmstadt, Germany). Neochlorogenic acid, cryptochlorogenic acid, isochlorogenic acid A, 1,5-di-*O*-caffeoylquinic acid, isoquercetin were obtained from Key Laboratory of Xinjiang Indigenous Medicinal Plants Resource Utilization, Xinjiang Technical Institute of Physics and Chemistry, Chinese Academy of Sciences. Luteolin, apigenin, hispidulin, galangin, isorhamnetin, narcissin, and isorhamnetin-3-*O*-glucose were purchased from Shanghai Pureone Biotechnology Co., Ltd (China). Chlorogenic acid, rutin, quercetin and kaempferol were purchased from China National Institute for the Control of Pharmaceutical and Biological Products.

### 2.2 Plant material

*M. Chamomile* was harvested in early July from Yili Kazakh Autonomous Prefecture, Xinjiang Uygur Autonomous Region, China and identified as Chamomile (*Matricarla chamomilla* L.) by Dr. Yonghe Li, Xinjiang Uygur Autonomous Region Hospital of Traditional Chinese Medicine. Fresh chamomile is stored at room temperature and naturally air-dried.

### 2.3 Active fraction preparation

The extract of *M. Chamomile* was prepared by reflux extraction method [16], and the ethanol concentration, time, and solid-liquid ratio were 70%, 1 h, and 1:10 (g/mL), respectively. The active fraction of *M. Chamomile* was enriched by AB-8 type macroporous adsorption resin, resin column diameter to height ratio of 1:6, drug solution mass concentration of 0.20 g/mL (equivalent to native drug), the velocity of absorption was 2 BV/h, after absorbed for 7BV, the column was washed by 1 BV water, 3BV 50% ethanol and 1BV 70% ethanol were used to elute the active components.

### 2.4 UHPLC-Q-Orbitrap-HRMS analysis

Chemical constituents of the active fraction of *M. Chamomile* were analyzed by UHPLC-Q-Orbitrap-HRMS. The dry active fraction (10 mg) was dissolved in methanol and filtered through 0.22 μm microporous membrane to obtain the sample solution (10 mg/mL). The ultra-high-performance liquid chromatography (UHPLC) system was conducted with Dionex Ultimate 3000 RSLC system (Thermo, Germany) which was equipped with the diode array detector (DAD). Chromatographic separation was achieved on an ACQUITY UPLC® BEH Shield RP18 column (2.1 × 100 mm, 1.7 μm, Waters, USA). The injection volume of sample was 5 μL and the flow rate was 0.3 mL/min. The column temperature was 35 °C and the scan wavelength ranged from 400 to 190 nm. The mobile phase was consisted of (solvent A) 0.1% (v/v) HCOOH and (solvent B) ACN. The gradient elution condition was followed: 0–9 min, 5% B; 9–11 min, 5–11% B; 11–36 min, 11% B; 36–64 min, 11–25% B; 64–70 min, 25–50% B.

The UHPLC system was coupled with Q-Exactive orbitrap high-resolution mass spectrometry (Thermo, Germany) equipped with electrospray ion (ESI) source. The parameters were as follows: negative ion −2.8 kV, positive ion 3.2 kV; sheath gas 40 arb; auxiliary gas 10 arb; gas curtain gas 35; ion source temperature 350 °C; capillary temperature 300 °C; collection range 100–1500 *m*/*z*; the fragment ion scanning range 50–1500 *m*/*z*; MS Resolution 70,000 FWHM (*m*/*z* 200); MS2 Resolution 17,600 FWHM (*m*/*z* 200). Data acquisition and analysis were performed using Xcailbur 4.0 software (Thermo, USA).

### 2.5 Animal

Forty clean grade SD (Sprague Dawley) rats, body mass 220 ± 20 g, male and female, were provided by the Experimental Animal Centre of Xinjiang Uygur Autonomous Region. During the experiments, rats were fed and watered freely and were housed in a standard clean grade animal laboratory at a room temperature of 20–25 °C and a 12 h light/dark cycle. The experimental procedures of the animal studies were permitted by the Institutional Animal Care and Use Committee of Xinjiang Medical University (approval number: IACUC-20210301-23).

#### 2.5.1 Establishment of asthma rat model

The asthmatic models of rats were established by ovalbumin (OVA). 40 SD rat animals were randomly divided into five groups: Group N (normal control group), Group M (OVA sensitization group), Group D (OVA + chamomile anti-asthmatic active site low dose group), Group Z (OVA + chamomile anti-asthmatic active site medium dose group) and Group G (OVA + chamomile anti-asthmatic active site high dose group), 8 animals in each group. OVA sensitization: Except for the normal control group, each rat was injected intraperitoneally with 1 mL OVA suspension (containing 1 mg OVA and 200 mg aluminium hydroxide gel) on days 1 and 8, and the normal control group was injected with the same dose of saline as a control.

OVA excitation: One week after the second sensitization, rats were placed in an ultrasonic nebulizer from 15th day, and stimulated by spraying saline aerosol containing 1% OVA with an ultrasonic nebulizer for 28 days, once a day and 30 min each time. The rats in the normal control group were nebulized with the same amount of saline. After stimulated, the rats were observed for signs of asthma attack such as irritability, sneezing, coughing, nasal scratching, incontinence, deepening of respiratory amplitude, wheezing and cyanosis, etc. Asthma modelling was considered successful.

#### 2.5.2 Pharmacological interventions

From day 43 to day 72, rats in each group received the following interventions: Group N: saline gavage; Group M: saline gavage; Group D: low dose gavage (0.06 g/kg dose) of the anti-asthmatic active site of chamomile; Group Z: medium dose gavage (0.12 g/kg dose) of the anti-asthmatic active site of chamomile; Group G: high dose gavage (0.18 g/kg dose) of the anti-asthmatic active site of chamomile. (0.18 g/kg dose).

#### 2.5.3 Behavioural observation

The OVA-induced allergic asthma rat model was established, and its behaviour was scored according to the “Asthma Attack Scale” to evaluate the presence or absence of symptoms of cyanosis, wheezing, shortness of breath, slow movement or twisting, agitation, etc. The asthma behavioural scale [17]: 0 points for normal or mild shortness of breath; 1 point for trembling or nodding; 2 points for coughing, marked shortness of breath, restlessness and cyanosis; 3 points for rhythmic retracted wheezing; 6 points for extreme respiratory distress with prostration or fall.

#### 2.5.4 Pulmonary function and level of total Ig E and glutathione peroxidase

Pulmonary function was measured by the Buxco Whole Volume Tracing System, USA (USA), according to reference [17]. 10% chloral hydrate intraperitoneal anesthesia, blood was taken from the abdominal aorta, centrifuged at 3500 r/min for 15 min, serum was taken and stored at −80 °C. The total IgE content in the serum of each group of rats was determined by rat ELISA kit, and the GSH-Px content was determined by Glutathione peroxidase (GSH-Px) kit.

#### 2.5.5 Collection bronchoalveolar lavage fluid (BALF) and eosinophil count

After blood taken from the abdominal aorta of the rat, the skin of the neck was cut longitudinally, the trachea was exposed, the chest cavity was opened to expose both lungs, the right main bronchus was ligated first; then a trocar needle was inserted into the trachea and ligated and fixed, 4 mL of sterile saline was instilled into the left lung, BALF was recovered after gentle massage of the lungs for 30 s, and the procedure was repeated three times (recovery rate 80%–90%). The supernatant was centrifuged at 3500 rmp for 5 min and stored at −80 °C. The sediment was resuspended in 0.5 mL Hanks' solution, and 0.2 mL suspension was used to evaluate eosinophil count in a small animal haemocytometer.

#### 2.5.6 Histopathological observation of lung

After collection of blood and BALF, the right lung was cut out and fixed in 10% neutral formaldehyde; the tissues were paraffin embedded and cut into sections at a thickness of 3 μm, then these were stained with hematoxylin-eosin (HE) to observe histopathology and inflammatory cell infiltration.

#### 2.5.7 Statistical analysis

SPSS® statistical software (version 11.0 for Windows) was used for analysis, and experimental data were expressed as mean ± standard deviation (mean ± SD). The behavioral observation scores of the rats in each group were compared by rank sum test; the enhanced respiratory intervals, eosinophil counts, serum IgE and GSH-PX levels of the rats were compared by one-way analysis of variance (ANOVA). *P* < 0.05 indicated that the differences were statistically significant.

## 3 Results and discussion

### 3.1 Optimization of Q-Orbitrap-HRMS conditions

**Fig. 1:**
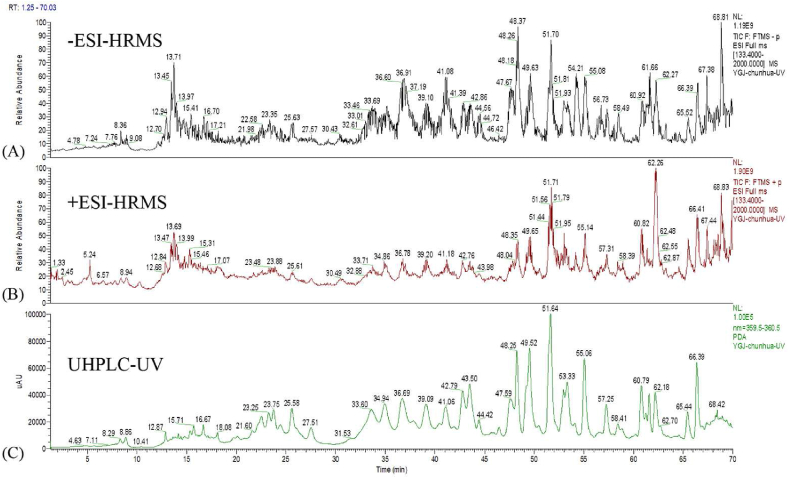
Total ion flow (TIC) of the active fraction of *M. chamomile* under negative mode (A) and positive mode (B), and UHPLC spectrum (C).

The mass spectra were evaluated in positive and negative ionization modes. The result showed that phenolic acids and flavonoids ionized well in the negative ionization mode, while alkaloids generated better signal intensities in positive ionization mode. In addition, there was little difference between positive and negative ion scanning of lignin compounds (Fig. 1). In the secondary mass spectrometry acquisition, the NCE was set to low, medium and high modes, which were 20, 40, 60 eV, respectively. Hence, rich fragment ion information of known and unknown compounds was collected widely.

### 3.2 Identification of chemical constituents

The active fractions of M. Chamomile were analyzed by UHPLC-Q-Orbitrap-HRMS. Considering the retention time (t_R_), precise mass-to-charge ratio (*m/z*), characteristic fragment ions, as well as available standards and previous literatures, Considering the retention time (t_R_), precise mass-to-charge ratio (*m/z*), characteristic fragment ions, as well as available standards and previous literature, 64 compounds were identified (Table 1), mainly including caffeoylquinic acid, flavonoids, coumarins and alkaloids.

(1) Caffeoylquinic acid

**Table 1:** Identification results of the active fraction of Chamomile by UHPLC-Q-Orbitrap-HRMS.

No.	t_R_ (min)	[M − H]^-^	[M+H]^+^	Error (ppm)	Formula	ESI-MS^2^ data (*m*/*z*)	Identification
**Caffeoylquinic acid**							
1	3.86	353.0882		4.2466	C_16_H_18_O_9_	191(100),179(30),173(10),135(60)	Neochlorogenic acid (3-*O*-caffeoylquinic acid
2	8.28	353.0863		−1.0792	C_16_H_18_O_9_	191(100),173(3),127(10)	Cryptochlorogenic acid (4-*O*-caffeoylquinic acid)
3	10.5	353.088		3.7188	C_16_H_18_O_9_	191(20),179(40),173(60),135(100)	Chlorogenic acid (5-*O*-caffeoylquinic acid)
4	26.68	677.1712		0.0134	C_31_H_34_O_17_	515(40),353(40),191(90),179(70),135(100)	di-*O*-caffeoylquinic acid-glucoside
5	33.26	515.1187		0.6824	C_25_H_24_O_12_	353(30),191(100),179(50),135(80)	1,3-di-*O*-caffeoylquinic acid
6	35.44	515.1193		1.827	C_25_H_24_O_12_	353(30),191(100),179(30),135(60)	3,4-di-*O*-caffeoylquinic acid (isochlorogenic acid B)
7	36.84	515.1196		2.4082	C_25_H_24_O_12_	353(20),191(100),179(40),135(90)	3,5-di-*O*-caffeoylquinic acid (isochlorogenic acid A)
8	47.6	515.1193		1.4038	C_25_H_24_O_12_	353(70),179(50),173(100),135,60)	4,5-di-*O*-caffeoylquinic acid (isochlorogenic acid C)
9	48.58	515.1186		0.5021	C_25_H_24_O_12_	353(50),191(60),179(80),173(100),135(80)	1,5-di-*O*-caffeoylquinic acid (isochlorogenic acid C)
10	56.05	529.1316		−4.5352	C_25_H_26_O_12_	367(10),353(50),193(5),191(80),179(70),173(100), 135 (90)	Caffeoyl-feruloyl-quinic acid
**Flavonoids**							
11	17.25	447.0924		0.4698	C_21_H_20_O_11_	357(60),327(100),299(50),285(30),133(45)	Lignocerebroside-8-*C*-glucoside
12	17.29	463.0877		1.4316	C_21_H_20_O_12_	300(100),283(5),255(5),227(5)	Quercetin-3-*O*-glucoside (isoquercitrin)
13	18.09	463.0878		1.5269	C_21_H_20_O_12_	301(100),283(5),255(5),227(5)	Chrysin
14	18.21	303.0508		3.0132	C_15_H_12_O_7_	285(50),217(5),151(5),125(100)	Dihydroquercetin
15	21.47	463.0876		1.2526	C_21_H_20_O_12_	301(100),271(40),255(10),243(5),151(30)	Quercetin-7-*O*-glucoside
16	22.15	477.0677		2.8037	C_21_H_18_O_13_	301(100),283(5),245(10),151(10)	Quercetin-*O*-glucuronide
17	22.19	609.1465		2.4655	C_27_H_30_O_16_	300(100),271(60),255(40),179(10),151(10)	Rutin
18	22.27	463.0875		0.8847	C_21_H_20_O_12_	301(100),271(30),255(10),243(5),151(10)	Quercetin-*O*-glucoside
19	22.55	477.0658		−1.1445	C_21_H_18_O_13_	301(100),283(5),245(10),151(20)	Quercetin-*O*-glucuronide isomers
20	22.67	463.0893		4.7835	C_21_H_20_O_12_	301(100),283(5),255(10),227(5),151(40)	Quercetin-*O*-glucoside isomers.
21	23.16	447.0919		−0.533	C_21_H_20_O_11_	285(50),284(100),227(10),137(30)	Kaempferol-3-*O*-glucoside (astragalin)
22	23.19	609.1464		2.4295	C_27_H_30_O_16_	300(100),271(60),255(40),179(10),151(10)	Rutin isomer
23	23.75	477.0678		3.1264	C_21_H_18_O_13_	301(100),283(5),245(10),151(20)	Quercetin-*O*-glucuronide isomers
24	24.54	447.0933		2.6068	C_21_H_20_O_11_	285(95),284(100),256(10),227(10),151(20)	Kaempferol-*O*-glucoside
25	24.63	461.0691		−4.921	C_21_H_18_O_12_	285(100),267(5),133(30)	Lignan-*O*-glucuronide
26	24.75	447.0936		3.2894	C_21_H_20_O_11_	285(100),284(95),256(10),227(10),133(20)	Luteoloside (Cynaroside)
27	25.55	447.0929		1.7877	C_21_H_20_O_11_	285(95),284(100),256(10),227(10),151(20)	Kaempferol 7-*O*-glucopyranoside
28	26.6	593.15109		1.6827	C_27_H_29_O_15_	285(100),269(5),227(10),133(20)	Lignan-*O*-glucosyl rhamnoside
29	31.94	623.16168		1.9261	C_28_H_32_O_16_	315(60),300(70),271(100),161(200,133(10)	Aquaporins
30	32.62	593.15109		0.9382	C_22_H_21_O_12_	315(50),299(100),271(70),243(20),133(20)	Isorhamnetin-3-*O*-glucoside
31	33.38	491.0838		2.1993	C_22_ H_20_ O_13_	315(60),300(50),271(100),255(20),243(80))	Methylquercetin-*O*-glucuronide
32	34.97	491.0831		2.3795	C_22_ H_20_ O_13_	315(60),300(40),271(100),255(30),243(80))	Methylquercetin-*O*-glucuronide isomer
33	38.11	431.0979		1.4713	C_21_H_20_O_10_	268(100),240(5),211(5),151(10)	Galangin-*O*-glucoside
34	39.19	431.0975		0.6144	C_21_H_20_O_10_	268(100),240(5),211(5),151(10)	Galangin-*O*-glucoside isomers.
35	39.66	577.1562		1.7672	C_27_H_30_ O_14_	269(100),225(5)	Apigenin-*O*-rhamnose-hexacoside.
36	47.95	607.1667		1.6173	C_28_H_32_ O_15_	299(40),284(30),255(100),227(70)	Methyl kaempferol-*O*-demethylhexoside
37	47.95	461.1078		−0.0171	C_22_H_22_ O_11_	446(20),299(5),283(100),255(80)	Methyl kaempferol-*O*-hexoside
38	51.31	505.0985		1.7912	C_23_H_22_O_13_	329(90),314(100),299(30),285(50),271(80),257(30), 243 (80)	Dihydroxyquercetin-glucuronide
39	51.7	491.119		1.2544	C_23_H_24_O_12_	47(20),329(10),313(100),298(50),270(80),242(20)	Dihydroxyquercetin-glucoside
40	52,32	301.0348		1.7155	C_15_H_10_O_7_	273(10),245(5),229(5),179(30),151(100)	Quercetin
41	53.58	285.0402		3.009	C_15_H_10_O_6_	241(10),217(5),151(20),133(100)	Luteolin
42	53.85	269.0435		3.2289	C_15_H_10_O_5_	225(10),201(10),151(20),117(60)	Apigenin
43	55.12	315.0509		3.3546	C_16_H_12_O_7_	300(100),283(5),243(5),137(20)	Methylquercetin
44	57.33	315.0508		2.9953	C_16_H_12_O_7_	300(80),271(100),255(40),243(30)	Isorhamnetin
45	60.88	269.0435		3.22895	C_15_H_10_O_5_	225(10),201(10),151(20),117(60)	Galangin
46	61.55	285.0402		3.2231	C_15_H_10_O_6_	257(10),239(5),211(20),185(5)	Kaempferol
47	62.27	299.0561		3.8696	C_16_H_12_O_6_	284(100),255(5),227(10),137(40)	Hispidulin
48	65.48	299.0562		4.1151	C_16_H_12_O_6_	284(40),255(100),227(90),211(10)	Methyl-cresol
**Coumarins**							
49	5.09		341.09	2.00581	C_15_H_15_O_9_	179(100),146(10), 133(30),119(20,91(15)	Dihydroxycoumarin-*O*-glucoside (Daphnoside)
50	7.87		179.04	−1.6791	C_9_H_6_O_4_	161(20), 146(10), 133(30),119(20),91(15)	Dithydroxycoumarin (Daphnetin)
51	8.86		193.05	2.0279	C_10_H_8_O_4_	178(40), 161(20),133(50), 91(5)	Hydroxy-methoxy-coumarin
52	15.43		193.05	1.7907	C_10_H_8_O_4_	178(50), 161(5), 133(60),91(10)	Hydroxy-methoxy-coumarin isomers
53	16.13		147.04	1.1011	C_7_H_6_O_2_	119(100), 91(80)	Coumarin
54	48.51		163.04	−2.1505	C_9_H_6_O_3_	145(50),135(100),117(70),107(20),89(80)	Hydroxycoumarin
55	49.97		325.09	−1.6887	C_15_H_16_O_8_	271(5),163(100),135(40),117(30)	Hydroxycoumarin glucoside
56	68.3		177.06	3.6747	C_10_H_8_O_3_	145(20), 131(100),117(20),103(50)	Methoxycoumarin
**Alkaloids**							
57	4.36		176.07	−1.6618	C_10_H_9_O_2_N	161(5),148(5),134(5),120(10),103(5)	Indole-acetic acid
58	4.6		146.06	−1.6516	C_9_H_7_ON	118 (40)	Indole-carbaldehyde
59	4.16		188.07	−2.3671	C_11_H_9_O_2_N	170(20),160(5),146(100), 118(80)	Acetylindole Formaldehyde
60	5.09		146.06	−1.9651	C_9_H_7_ON	118(20),91(5)	Indole-carbaldehyde isomers.
61	5.17		188.07	1.1216	C_11_H_9_O_2_N	170(20), 146(100), 118(80)	Acetylindole Formaldehyde isomers
**Other compounds**							
62	1.05	191.0188		1.4829	C_6_H_8_O_7_	111 (100)	Citric acid
63	8.12	179.0343		2.3266	C_9_H_9_O_4_	135 (100)	Caffeic acid
64	8.36	191.0553		1.7225	C_7_H_12_O_6_	176(70),148(30),111(5)	Quinic acid

Caffeoylquinic acid is composed of caffeic and quinic acids. Generally, there are more substitution at 3,4,5 positions on quinic acid, but less substitution at 1 position. The caffeoylquinic acid compound has a good respone in the ESI negative ion mode. The secondary mass spectra showed mainly characteristic fragment ions such as *m/z* 191 [quinic acid-H]-, *m/z* 179 [caffeic acid-H]- and *m/z* 173 [quinic acid–H_2_O–H]- [18]. Ten caffeoylquinic acid compounds were identified in the active fraction of *M. Chamomile*.

**Fig. 2:**
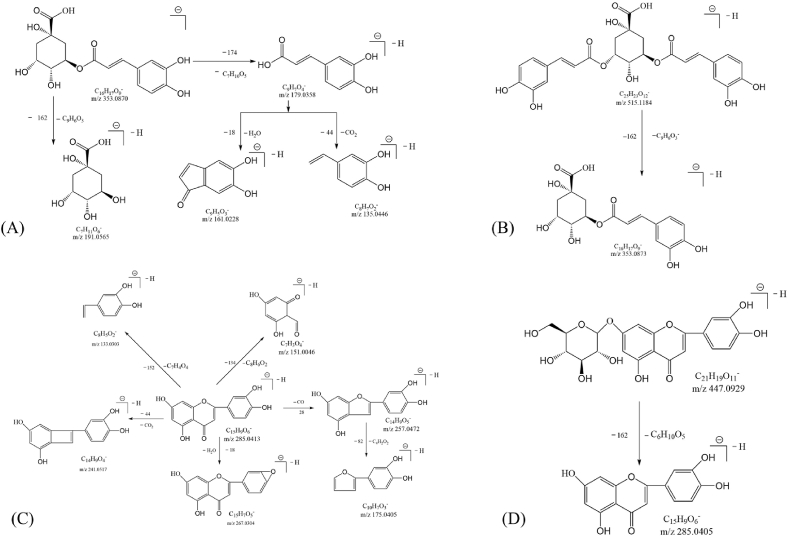
Possible mass spectrometric cleavage rule of Chlorogenic acid (A), Isochlorogenic acid A (B), Luteolin (C), and Cynaroside (D).

Compound 1 (t_R_ = 3.86 min), compound 2 (t_R_ = 8.28 min) and compound 3 (t_R_ = 10.53 min) all showed molecular ion *m*/*z* 353 [M − H]^-^. In secondary mass spectra, they all had characteristic fragment ions at *m/z* 191, 179, and 173. Compounds 1, 2 and 3 were identified as neochlorogenic acid (3-*O*-caffeoylquinic acid), cryptochlorogenic acid (4-*O*-caffeoylquinic acid) and chlorogenic acid (5-*O*-caffeoylquinic acid), respectively, based on the retention times and the agreement of the fragment ions with the standards [19]. The cleavage rule of chlorogenic acid was shown in Fig. 2A.

Compound 4 (t_R_ = 26.68 min) showed a molecular ion peak at *m/z* 677 [M − H]^-^. In the secondary mass spectrum, fragment ions of *m/z* 515 [M − 162]^−^indicated that the glucose were lost from molecular ion. Also, di-*O*-caffeoylquinic acid of characteristic fragment ions *m/z* 353, 191, 179, and 135 were observed. Therefore, it was presumed that compound 4 was di-*O*-caffeoylquinic acid-glucoside based on the cleavage characteristics of the compound [20].

Compound 5 (t_R_ = 33.26 min), compound 6 (t_R_ = 3 5.44 min), compound 7 (t_R_ = 36.84 min), compound 8 (t_R_ = 47.60 min) and compound 9 (t_R_ = 48.58 min) had same the molecular ion at *m*/*z* 515 [M − H]^-^. The fragment ion of secondary mass spectra at *m/z* 353, suggesting that these compounds were decaffeoylquinic acid. Compounds 5, 6, 7, 8 and 9 were identified as 1,3-di-*O*-caffeoylquinic acid, 3,4-di-*O*-caffeoylquinic acid (isochlorogenic acid B), 3,5-di-*O*-caffeoylquinic acid (isochlorogenic acid A), 4,5-di-*O*-caffeoylquinic acid (isochlorogenic acid C), and 1,5-di-*O*-caffeoylquinic acid (isochlorogenic acid C), based on the retention times and the cleavage characteristics of these compounds with the standards. C) [[21], [22], [23]]. The cleavage rule of isochlorogenic acid A was shown in Fig. 2B.

Compound 10 (t_R_ = 56.05 min) showed a molecular ion peak at *m/z* 529 [M − H]^-^. Characteristic fragment ions of the secondary mass spectra were observed at *m/z* 367 [feruloylquinic acid-H]^-^, *m/z* 353 [caffeoylquinic acid-H]^-^, and *m/z*193 [ferulic acid-H]^-^, *m/z* 191 [quinic acid-H]^-^, *m/z* 179 [caffeic acid-H]^-^, *m/z* 173 [quinic acid–H_2_O–H]^-^, and *m/z* 135 [caffeic acid-CO_2_]^-^. According to the similarity of the mass spectral cleavage rules and the previous literature [24], the compound was identified as caffeoyl-feruloyl-quinic acid.

(2) Flavonoids

According to the fragmentation pattern and previous study, 38 flavonoids were identified from the active fractions of *M. Chamomile*. Among these compounds, were linked to the flavonoid parent nucleus via C-O bonds, with the exception of compound 11.

Compounds 11 (t_R_ = 17.25 min), 25 (t_R_ = 24.63 min), 26 (t_R_ = 24.75 min), 28 (t_R_ = 26.60 min) and 41 (t_R_ = 53.38 min) presented molecular ions at *m/z* 447, 461, 447, 593 and 285 [M − H]^-^, respectively. The MS^2^ spectrum showed the fragment ions of compound 41 at were *m/z* 241, 271, 151, and 133. According to retention time and standard, compounds 41 was identified as luteolin, the cleavage rule was shown in Fig. 2C, [25]. The base fragment ion peak at *m/z* 285 was shown in the secondary mass spectra of compounds 11, 25, 26 and 28, which was the characteristic fragment ion of luteolin. Therefore, Therefore, this confirmed the mother nucleus of these compounds was lignocerebrosides. Based on the retention times of compounds 11 and 26 and the fragment ions with the standards, these two compounds were identified as lignocerebroside-8-C-glucoside and lignocerebroside (Cynaroside, Fig. 2D), respectively. Compound 25 had molecular ion at *m/z* 285 [M-H-176]^-^ could be attributed to the loss of glucuronic acid, and compound 28 had molecular ion at *m/z* 285 [M-H-162-146]^-^ could be attributed to the loss of a glucose and rhamnose. Therefore, compounds 25 and 28 are presumed to be lignan-*O*-glucuronide and lignan-*O*-glucosyl rhamnoside, respectively. However, the exact position is needed to be confirmed further.

Compounds 12 (t_R_ = 17.29 min), 13 (t_R_ = 18.09 min), 15 (t_R_ = 21.47 min), 18 (t_R_ = 22.27 min) and 20 (t_R_ = 22.67 min) had same molecular ion peaks at *m/z* 463 [M − H]^-^. The fragment ion at *m/z* 301 and 300 indicated that these compounds contain glucose group. The fragment ions at *m*/*z* 255, 227, and 151 indicate that the parent nucleus was a quercetin glycogen. Therefore, these compounds are presumed to be quercetin-*O*-glucoside and its isomers. Based on the above mass spectral information and the agreement of retention times with the standards, compounds 12, 13 and 15 were deduced to be quercetin-3-*O*-glucoside (isoquercitrin), chrysin, and quercetin-7-*O*-glucoside, respectively [24]. The position of the sugar was probably 3′ or 4′ glucoside, but this could not be confirmed based on the current mass spectrometry information and was tentatively identified as quercetin-*O*-glucoside and its isomers.

Compounds 16 (t_R_ = 22.15 min), 17 (t_R_ = 22.19 min), 19 (t_R_ = 22.55 min), 22 (t_R_ = 23.19 min), 23 (t_R_ = 23.75 min) and 40 (t_R_ = 52.32 min) had an [M − H]^-^ ion at *m/z* 477, 477, 609, 477, 609 and 301, respectively. 477, 609, 477, 609 and 301 [13,26,27]. Compared with retention times and mass spectral fragmentation information of the standards, compounds 17 and 40 were identified as rutin and quercetin, respectively. The fragment ion of compounds 22 was similar to that of compound 17, and it was tentatively identified as the rutin isomer. Compounds 16, 19 and 23 had same fragment ion at *m/z* 301 [M-H-176]^-^, indicating that all were missing a glucuronide. The fragment ions at *m/z* 255, 227, and 151 indicated that the parent nucleus is a quercetin. Compounds 16, 19 and 23 are presumed to be quercetin-*O*-glucuronide and its isomers, respectively, based on the characteristic ions fragment and previous literature [28,29].

Compounds 21 (t_R_ = 23.16 min), 24 (t_R_ = 24.54 min) and 27 (t_R_ = 25.55 min) had same molecular ion peaks at *m/z* 447 [M − H]^-^. The fragment ions at *m/z* 284 [M-H-H-162]^-^ or 285 [M-H-162]^-^ indicated the loss of a glucose. The characteristic ions of kaempferol were *m/z* 255, 227, 163, and 151. Therefore, these compounds presumed to be kaempferol-*O*-glucoside and its isomers based on the above mass spectral information and reference [30]. According to retention time with the standard, compound 21 was identified as kaempferol-3-*O*-glucoside (astragalin).

Compared with fragment information of mass spectrum and the standards, compounds 29 (t_R_ = 31.94 min), 30 (t_R_ = 32.62 min), 42 (t_R_ = 53.85 min), 44 (t_R_ = 57.33 min), 45 (t_R_ = 60.88 min), 46 (t_R_ = 61.55 min) and 47 (t_R_ = 62.27 min) were identified as aquaporins, isorhamnetin-3-*O*-glucoside (apigenin) [25], isorhamnetin, galangin [31], kaempferol and hispidulin [32].

Compounds 31 (t_R_ = 33.38 min), 32 (t_R_ = 34.97 min) and 43 (t_R_ = 55.12 min) presented the molecular ion at *m/z* 491, 491, and 315, respectively. There were more abundant fragment ions at *m/z* 315 and 300 in the secondary mass spectra. However, the retention time of compound 43 was not consistent with that of isorhamnetin. Therefore, compounds 31, 32 and 43 were inferred to be methylquercetin-*O*-glucuronide, methylquercetin-*O*-glucuronide isomer and methylquercetin, respectively, based on the available mass spectral information and similarities in the prior literature.

Compounds 33 (t_R_ = 38.11 min) and 34 (t_R_ = 39.19 min) had same molecular ion peaks at *m/z* 431 [M − H]^-^. The fragment ion at *m/z* 268 as the base peak in the MS^2^ spectrum, indicating the loss of a glucose. The fragmentation information at *m/z* 268, 240, 211, 151 were consistent with galangin, hence these two compounds are galangin-*O*-glucoside and its isomers.

Compound 35 (t_R_ = 39.66 min) had molecular ion 577 [M − H]^-^ and showed fragment ion at *m/z* 269 as the base peak in the MS^2^ spectrum, indicating the loss of a rhamnose and glucose. The other fragment ions were *m/z* 255, 117, 107, and 149, suggesting that the molecular parent was apigenin. Therefore, based on the above information and previous literature reports, the compound was deduced to be apigenin-*O*-rhamnose-hexacoside.

Compounds 36 (t_R_ = 47.95 min), 37 (t_R_ = 48.19 min) and 48 (t_R_ = 65.48 min) showed molecular ion peaks at *m/z* 607, 461 and 299, respectively. According to characteristic fragment ions at *m/z* 284 [M − CH_3_]^-^, 255 [M − CH_3_ –CO–H]^-^, 227 [M–CH_3_–2CO-H]^-^ and 211 [M–CH_3_– CO-CO_2_-H]^-^, compound 48 was identified as methyl kaempferol. Based on the above mass spectral information and references, compounds 36 and 37 had same fragment ion at *m/z* 299, indicating the loss of desmethyl glucose and glucose, respectively. Therefore, compounds 36 and 37 were identified as methyl kaempferol-*O*-demethylhexoside and methyl kaempferol-*O*-hexoside, respectively.

Compounds 38 (t_R_ = 51.31 min) and 39 (t_R_ = 51.70 min) have molecular ion [M − H]^-^ at *m/z* 505 and 491, respectively. In the MS^2^ spectrum, the characteristic ions of dihydroxyquercetin at *m/z* 329, 314, 299 and 271. Based on the mass spectral information and previous literature reports, the compounds were tentatively identified as dihydroxyquercetin-glucuronide and dihydroxyquercetin-glucoside.

(3) Coumarins

Coumarins are correspondingly highly ionized in the positive ion mode, and eight coumarins were identified from the active fraction of *M. Chamomile*.

Compounds 49 (t_R_ = 5.09 min) and 50 (t_R_ = 7.87 min) presented the molecular ion at *m/z* 341 and 179 [M+H]^+^, respectively. Compounds 50 had characteristic fragment ions of coumarins at *m/z* 119 and 91 in the MS^2^ spectrum. Based on mass spectral fragment information and retention times consistent with the standards, compounds 49 and 50 were deduced to be dihydroxycoumarin-*O*-glucoside (daphnoside) and dihydroxycoumarin (daphnetin), respectively [33].

Compounds 51 (t_R_ = 8.86 min) and 52 (t_R_ = 15.53 min) had same molecular ion peaks at *m*/*z* 193. In MS^2^ spectrum, fragment ions *m/z* 178 [M + H-CH_3_]^+^, *m/z* 146 [M + H-CH_3_OH]^+^, and the more abundant *m/z* 133 [M + H-CH_3_OH-CO]^+^ can be observed. 133 [M + H-CH3OH-CO]^+^. Based on the above mass spectrometric information and previous literature, the compound was identified as a hydroxy-methoxy-coumarin and its isomers [34].

Compounds 53 (t_R_ = 16.13 min), 54 (t_R_ = 48.51 min), 55 (t_R_ = 49.97 min) and 56 (t_R_ = 68.30 min) showed the molecular ion *m/z* 147, 163, 325 and 177 [M+H]^+^, respectively. The fragment ion at *m*/*z* 119 and 91 as well as retention time were consistent with the standard, hence compound 53 was identified as coumarins. The MS^2^ spectra of compounds 54, 55 and 56 all had fragment ions at *m/z* 145, suggesting that the parent nucleus was coumarin. Based on the mass spectral information and literature reports, compounds 54, 55 and 56 were deduced to be hydroxycoumarin, hydroxycoumarin (inocyanin) and methoxycoumarin, respectively [35].

(4) Alkaloids

Five indole alkaloids were identified in the active fraction of *M. Chamomile*, and fragment ions of MS^2^ spectra at *m/z* 117 or 118, representing the parent nucleus was the indole alkaloids. Under higher collision energies, the parent nucleus of the indole alkaloids produce characteristic fragment ions at *m/z* 91 and 77. These characteristic fragment information can be used for the determination of indole-like alkaloids [36]. Compound 57 (t_R_ = 4.36 min) represented molecular ion *m/z* 176 [M+H]^+^, and the structural formula was C_10_H_10_O_2_N. The fragment ion at *m/z* 117 [M + H-59]^+^ was observed in the MS^2^ spectrum, indicating the loss of the acetic acid. Therefore, the compound was deduced to be indole-acetic acid.

Compounds 58 (t_R_ = 4.60 min) and 60 (t_R_ = 5.37 min) had molecular ion peaks [M+H]^+^ at *m/z* 146, and the structural formula was C_9_H_6_ON. The fragment ion at *m/z* 118 [M + H-28]^+^ was observed in the MS^2^ spectra, indicating the loss of a formyl group. The fragment ions at *m/z* 91 and 77 were the characteristic fragment information of the indole-like parent nucleus. Therefore, compounds 58 and 60 are inferred to be indole-carbaldehyde and its isomers.

Compounds 59 (t_R_ = 4.16 min) and 61 (t_R_ = 5.09 min) showed same molecular ion peak at *m/z* 188 [M+H]^+^, with a structural formula of C_11_H_10_O_2_N. The fragment ion at *m/z* 146 [M + H-42]^+^ was observed in the MS^2^ spectrum, suggesting that the structure contained an acetyl group; and the fragment ion at *m/z* 118 [M + H-42-28]^+^, suggesting further loss of the formyl group. Also, indole-like characteristic fragment ions such as *m*/*z* 91, 77 were observed. Based on the above information, compounds 59 and 61 were inferred to be acetylindole formaldehyde and its isomers, respectively.

(5) Other compounds

Compounds 62 (t_R_ = 1.05 min) and 64 (t_R_ = 8.36 min) showed same molecular ion peaks at *m/z* 191 [M − H]^-^, and molecular formulas were C_6_H_9_O_7_ and C_7_H_13_O_6_, respectively. Based on the retention time and fragmentation information with the standard, compound 62 and 64 were dentified as citric acid and quinic acid [37,38].

Compound 63 (t_R_ = 8.12 min) showed a molecular ion peak [M − H]^-^ at *m/z* 179, and fragment ions were observed in the secondary mass spectrum at *m/z* 135, indicating the loss of CO_2_. Based on retention time and mass spectral information consistent with the standard, it was confirmed as caffeic acid [39].

### 3.3 Behavioural observations of the rats in each group

**Fig. 3:**
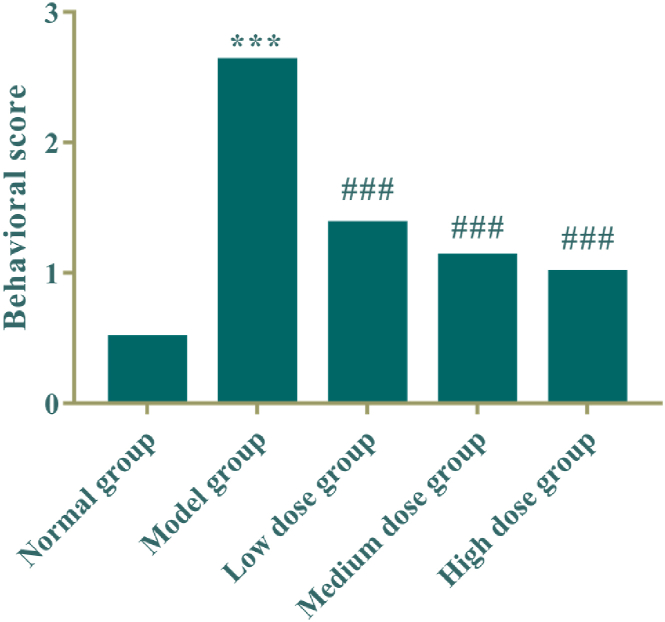
Behavioral score of rats (means, n = 8). Compared with the normal control group, ***P < 0.005; Compared with the model group, ^###^*P* < 0.005.

The results are shown in Table 2 and Fig. 3 that the behavioural scores of the model group were significantly higher than those of the normal control (*P* < 0.005); administration group was reduced in turn compared with the model group in a dose-dependent manner (*P* < 0.005).

**Table 2:** Behavioral score of rats (means, n = 8).

group	Normal group	Model group	Low dose group	Medium dose group	High dose group
1	0	3	2	1	1
2	1	3	1	0	1
3	1	2	1	2	0
4	1	2	2	1	2
5	0	2	1	1	0
6	1	3	1	2	1
7	0	3	2	1	2
8	0	3	1	1	1
Mean value	0.5	2.625	1.375	1.125	1

### 3.4 Effect of the active fraction of *M. Chamomile* on the enhanced respiratory intervals (Penh) in asthmatic rats

The results were shown in Table 3 that Penh was significantly higher in the model group compared to the normal control group (*P* < 0.01); compared with the model group, Penh of administration groups was reduced significantly in rats (*P* < 0.01).

**Table 3:** Effect of active fraction of *M. Chamomile* on Penh changes (mean ± SD, n = 8).

Group	Dose/g·kg^−1^·d^−1^	Penh
Normal group	\	0.437 ± 0.064
Model group	\	0.643 ± 0.140**
Low dose group	0.06	0.492 ± 0.073^##^
Medium dose group	0.12	0.474 ± 0.090^##^
High dose group	0.24	0.466 ± 0.079^##^

### 3.5 The effect of the active fraction of *M. Chamomile* on serum IgE in rats with bronchial asthma

**Fig. 4:**
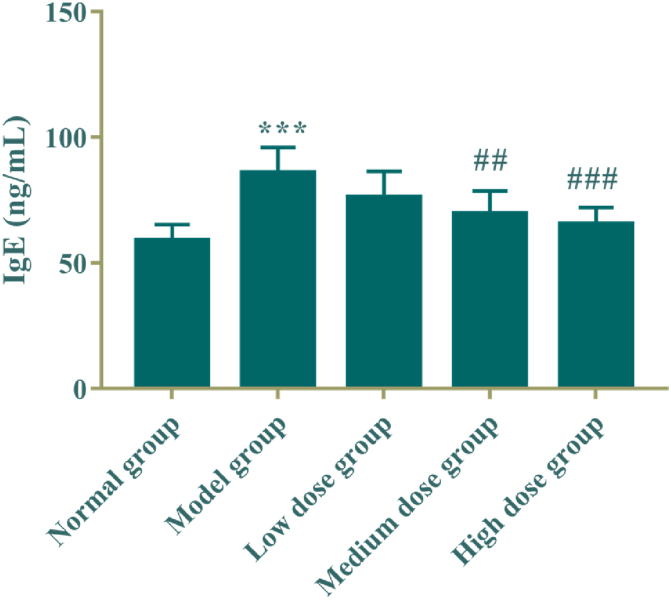
Effect of active fraction of *M. Chamomile* on serum IgE of bronchial asthma rats (mean ± SD, n = 8). Compared with the normal control group, ***P < 0.005; Compared with the model group, ^##^*P* < 0.01, ^###^*P* < 0.005.

The results were shown in Table 4 and Fig. 4 that the serum IgE level was significantly increased in the model group (*P* < 0.005), compared with the normal control group; and, compared with the model group, the middle and high dose groups of the active fraction of *M. Chamomile* could significantly reduce the IgE level in rats (*P* < 0.01, *P* < 0.005).

**Table 4:** Effect of active fraction of *M. Chamomile* on serum IgE of bronchial asthma rats (mean ± SD, n = 8).

Group	Dose/g·kg^−1^·d^−1^	IgE (ng/mL)
Normal group	\	58.81 ± 6.48
Model group	\	85.83 ± 10.06***
Low dose group	0.06	76.08 ± 10.31
Medium dose group	0.12	69.61 ± 9.10^##^
High dose group	0.24	65.27 ± 6.75^###^

### 3.6 Effect of the active fraction of *M. Chamomile* on eosinophil counts in BALF of rats with bronchial asthma

**Fig. 5:**
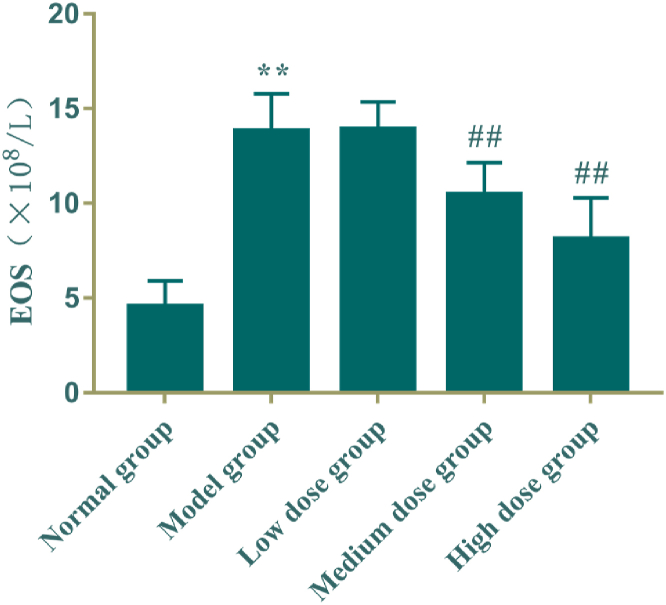
Effect of active fraction of *M. Chamomile* on eosinophils count in bronchoalveolar lavage fluid (BALF) in rats (mean ± SD, n = 8). Compared with the normal control group, **P < 0.01; Compared with the model group, ^##^*P* < 0.01.

The results were shown in Table 5 and Fig. 5 that the eosinophil count in BALF of the model group was significantly higher than that of the normal control group (*P* < 0.01); the eosinophil count was reduced significantly in BALF of rats in the middle and high dose groups of the active fraction of *M. Chamomile*, compared with the model group (*P* < 0.01).

**Table 5:** Effect of active fraction of *M. Chamomile* on eosinophils count in bronchoalveolar lavage fluid (BALF) in rats (mean ± SD, n = 8).

Group	Dose/g·kg^−1^·d^−1^	EOS（ × 10^8^/L）
Normal group	\	4.56 ± 1.34
Model group	\	13.80 ± 1.99**
Low dose group	0.06	13.90 ± 1.46
Medium dose group	0.12	10.46 ± 1.70^##^
High dose group	0.24	8.11 ± 2.18^##^

### 3.7 Effect of the active fraction of *M. Chamomile* on serum glutathione peroxidase (GSH-Px) in rats with bronchial asthma

**Fig. 6:**
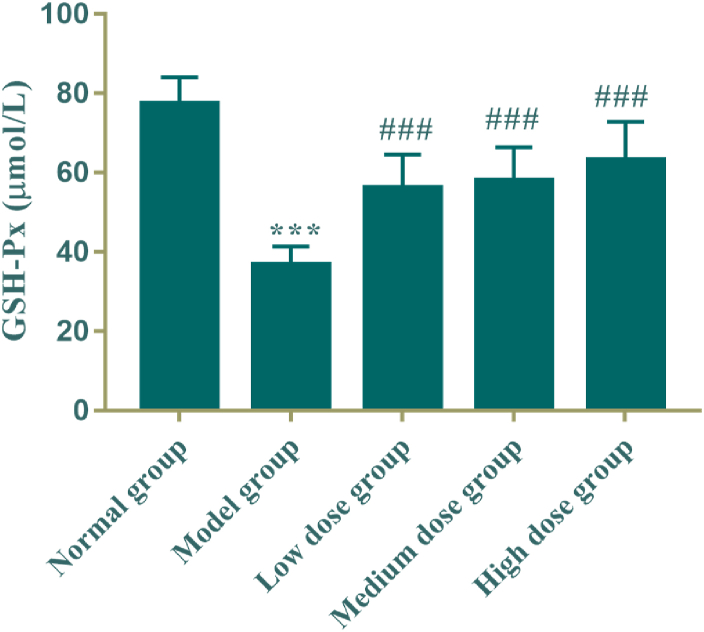
Effect of active fraction of *M. Chamomile* on serum GSH-Px in bronchial asthma rats (mean ± SD, n = 8). Compared with the normal control group, **P < 0.005; Compared with the model group, ^###^*P* < 0.005.

The results were shown in Table 6 and Fig. 6 that the serum GSH-Px levels in model group were significantly lower than that of the normal control group (*P* < 0.005); and compared with the model group, the serum GSH-Px levels of administration group was increased significantly in a dose-dependent manner (*P* < 0.005).

**Table 6:** Effect of active fraction of *M. Chamomile* on serum GSH-Px in bronchial asthma rats (mean ± SD, n = 8).

Group	Dose/g·kg^−1^·d^−1^	GSH-Px (umol/L)
Normal group	\	77.35 ± 6.81
Model group	\	36.89 ± 4.56***
Low dose group	0.06	56.22 ± 8.40^###^
Medium dose group	0.12	58.07 ± 8.40^###^
High dose group	0.24	63.22 ± 9.63^###^

### 3.8 Histopathological analysis of the lungs

**Fig. 7:**
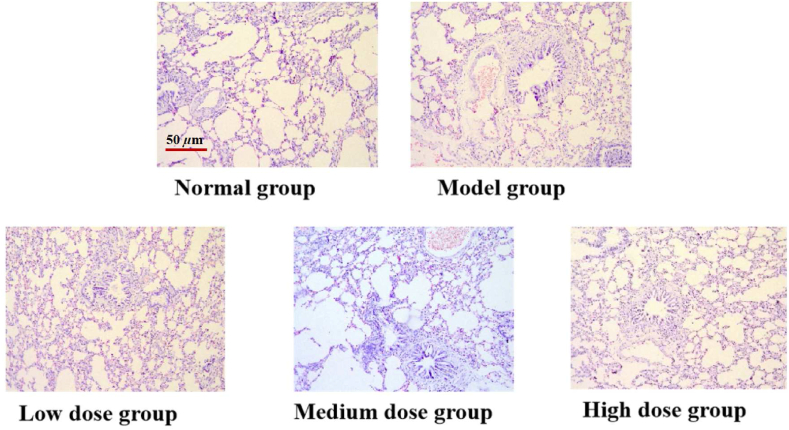
The microscopic structure of lung histopathology of rats with HE staining. The pictures are 200× (scale bars = 50 μm).

The results were shown in Fig. 7. The lung tissue of the rats in the blank group was basically normal in morphology, with no inflammatory cell infiltration around the bronchi and blood vessels, while occasional secretions and exfoliated cells in the lumen of the bronchi. In the model group, inflammatory cells were seen around the bronchi and blood vessels, and blood vessels were congested, and eosinophilic infiltration was seen in the lung tissue of rats. In the low dose group, inflammatory cell infiltration and vascular congestion were seen around the bronchi and blood vessels of the lung tissue of rats, and a small amount of eosinophil infiltration was seen in the lung tissue of rats. The degree and scope of inflammatory cell infiltration such as lymphocytes and eosinophils around the bronchi and blood vessels of lung tissue in the middle and high dose groups of Chamomile Anti-Asthma Active Part were significantly reduced compared with the model group.

## 4 Discussion and conclusion

Asthma is a life-threatening chronic airway disease. There are about 300 million people suffering from asthma in the world, and this number is increasing at an alarming level, reaching an annual asthma mortality rate of more than 250,000. T cells associated with asthma are considered to be the key pathogenic factors of inflammatory response in asthma, which release various Inflammatory cytokine, such as interleukin (IL-2, IL-4, IL-5, IL-10, IL-13, etc.), tumor necrosis factor-α (TNF-α), interferon-γ (IFN-γ) and specific allergens produced by activated B lymphocytes [40]. Goblet cells [41] produce excessive mucus, bronchospasm, reversible airway obstruction and airway inflammation caused by eosinophil infiltration, in addition, it will increase airway smooth muscle contraction and mast cell degranulation to release histamine, causing a series of asthma symptoms. In addition, these inflammatory cells secrete reactive oxygen species (ROS) and induce oxidative stress and inflammatory process, which is considered as the main potential cause of asthma [42].

In this study, UHPLC-Q-Orbitrap-HRMS coupling technique was used to the separation and identification of the components in the active fraction of *M. Chamomile*. A rapid and effective systematic identification method of chemical components of chamomile was established the separation and identification of the components in the active fraction of *M. Chamomile*. The chromatographic peaks of quasi-molecular ions were obtained, 64 compounds were identified by combining the standards, related literatures and secondary mass spectrometry fragments, including 10 caffeoyl quinic acid compounds, 38 flavonoids, 8 coumarins, 5 alkaloids and 3 other compounds. This study demonstrated that the active fraction of *M. Chamomile* contained abundant polyphenol compounds, especially flavonoids, which have been said to account for most of the antioxidant activities and anti-inflammatory effects of plant extracts [6,43,44]. Previous studies found that luteolin, apigenin, quercetin, kaempferol and their related compounds had significant inhibitory effect on IL-4 synthesis and TNF-α [45]; luteolin, quercetin and baicalein can respond to high affinity IgE receptor through inhibit the secretion of granulocyte macrophage-colony stimulating factor (GM-CSF) [46]. Flavonoids are antioxidants and anti-allergic nutrients that inhibit the release of chemical mediators and synthesis of Th2 type cytokines, several epidemiological studies suggest that it is beneficial for asthma to increase in flavonoid intake [47]. Chlorogenic acid can inhibite pulmonary eosinophilia, IgE production, and Th2-type cytokine production in the lung of mice with allergic asthma induced by ovalbumin [48]. Coumarin and coumarin-related compounds are also potential anti-inflammatory agents due to inhibition of cyclic nucleotide phosphodiesterases that generate cAMP and cGMP increasing [49]. These previous researchs indicated that polyphenol compounds from the active fraction of *M. Chamomile* were effective components against asthma, especially flavonoids were the important bioactive components, mainly including luteolin, quercetin, kaempferol and their related compounds. Hence, the identification results provide a basis for clarifying the active fraction of *M. Chamomile* and the quality control of related new drug of anti-asthma development process.

Furtherly, the OVA-induced allergic asthma rat model was established for the purpose of investigating the anti-asthma effect of the active fraction of *M. Chamomile.* The results showed that the number of EOS in Penh and bronchoalveolar lavage fluid (BALF) in the active fraction of *M. Chamomile* was significantly lower than that in the model group. IgE has been the immunoglobulin traditionally linked to an allergic response in conditions such as asthma; and enzyme-linked immunosorbent assay (ELISA) was used to detect the serum IgE level. The active fraction of *M. Chamomile* can significantly reduce the serum IgE level of asthmatic rats induced by OVA. In addition, compared with the asthma model group, the active fraction of *M. Chamomile* significantly increased glutathione peroxidase (GSH-Px) in the serum and ameliorated lung injury significantly. The results suggested that the active fraction of *M. Chamomile* had inhibitory effects on airway inflammation, hyperresponsiveness and oxidative stress in asthmatic rat.

Overall, combing with retention time, mass data, standards and information reported previously, 64 compounds were identified from the active fraction of *M. Chamomile* by UHPLC-Q-Orbitrap-HRMS technology, including 10 caffeoylquinic acids, 38 flavonoids, 8 coumarins, 5 alkaloids and 3 other compounds. Flavonoids were the important bioactive components of *M. Chamomile* against asthma, especially luteolin, quercetin, kaempferol and their related compounds. And, the active fraction of *M. Chamomile* possessed potent anti-inflammatory and anti-oxidative effects to exerts protective effects against OVA-induced asthmatic symptoms. Hence, *M. Chamomile* could be used in the future treatment of asthma, and future research is required to explore the underlying mechanisms.

## Author

Conceptualization, X.X. and H.A.A.; methodology, Q.L. R. A. G.X, and X.K; software, X.X.; validation, R.A.; formal analysis, Q.L. and R. A.; investigation, Q.L. R. A. G.X, X.K and F.Z; resources, X.X. F.Z and H.A.A.; data curation, R.A.; writing—original draft preparation, Q.L. R.A. G.X, and X.K; writing—review and editing, X.X. and H.A.A.; visualization, X.X.; supervision, X.X. and H.A.A.; project administration, H.A.A.; funding acquisition, Q.L All authors have read and agreed to the published version of the manuscript.

## Funding

This work was supported by 10.13039/501100015310Xinjiang Uygur Autonomous Region Natural Science Fund (2021D01E32).

## Declaration of competing interest

The authors declare that they have no known competing financial interests or personal relationships that could have appeared to influence the work reported in this paper.
